# Exploring correlation between social determinants and overweight/obesity in children and youths with epilepsy

**DOI:** 10.3389/fped.2022.897333

**Published:** 2022-10-21

**Authors:** Jie Yang, Fang Chen

**Affiliations:** Department of Pediatrics, Affiliated Hospital of North Sichuan Medical College, Nanchong, China

**Keywords:** children, epilepsy, obesity, education, poverty level

## Abstract

**Aim:**

This study aimed to explore the correlation between social determinants and overweight and obesity in children and youths with epilepsy.

**Methods:**

The study data were derived from the National Survey of Children's Health (NSCH) 2016–2017 and 2018–2019, a cross-sectional sample of young people aged 10–17. Three groups participated by weight: 423 in body mass index (BMI) 5th–84th group (normal weight), 108 in BMI 85th–94th group (overweight), and 124 in BMI ≥ 95th group (obesity). Multivariate ordinal logistic regression analyses were conducted. The three subgroups were divided to explore the correlation between social determinants and overweight and obesity.

**Results:**

A total of 655 children were included. After adjusting for sex, age, race, use of cigarets, cigars, or pipe tobacco inside, afterschool activity, and physical activity, children in poor physical condition reported by their parents [OR = 1.573 (95% CI, 1.164–2.125)] were associated with overweight/obesity. There were negative correlations between parents with higher education and overweight/obesity, especially in children and youths with previous or current epilepsy groups. Also, overweight/obesity was correlated with the 200%–400% family poverty level (FPL) [the adjusted odds ratio (OR) = 0.156 (95% CI, 0.028–0.876)] and above 400% FPL [the adjusted OR = 0.121 (95% CI, 0.023–0.641)] in children and youths with mild symptoms of epilepsy, and above 400% FPL [the adjusted OR = 0.103 (95% CI, 0.023–0.460)] in children with moderate to severe epilepsy.

**Conclusion:**

Poor physical conditions were correlated with obesity in childhood epilepsy. Parents with higher education and FPLs were negatively correlated to childhood obesity. Therefore, this study was intended to advocate for a greater emphasis on BMI for children and youths with epilepsy in families with lower-educated and low-income groups.

## Introduction

Globally, obesity rates are high among children and youths, and recent data show that it is on the rise (1, 2). Since the mid-1970s, more than 1 billion adults have been overweight, 650 million adults, and 124 million children and youths are obese (3). Obesity can cause a variety of comorbidities, these diseases will continue into adulthood, and may shorten the child's lifespan (4). Recent studies confirm the high comorbidities of childhood obesity and epilepsy (5), and children with epilepsy are more likely to be obese or overweight than normal children (6). Epilepsy is a chronic, non-communicable disease of the brain that is diagnosed in an estimated 5 million people worldwide each year (7). Both obesity and epilepsy are a serious threat to the healthy growth of children, suggesting that it is very important and necessary to intervene in the obesity status of children with epilepsy as early as possible.

Previous studies assessed the independent relationship between sociodemographic and economic factors of childhood obesity in the United States and showed that the prevalence of obesity in children and youths has increased in certain races/ethnicities and those with low socioeconomic status (8–11). Moreover, it is quite common to find children with epilepsy in low-income and middle-income countries, with an estimated incidence of 21–41 per 1,000 older children (6–9 years old) (12). Currently, there was a study on the relationship between overweight/obesity and psychosocial functioning (aggressive/aggressive behavior, social dysfunction) in children and youths with epilepsy (13). However, few studies have shown the relationship between the perspective of social, family, or child growth factors and obesity in children and youths with epilepsy.

We hypothesized a correlation between social determinants and overweight and obesity in children and youths with epilepsy. Herein, this study aimed at exploring the correlation between social determinants and overweight/obesity in children and youths who have or are currently suffering from epilepsy in the United States using nationally representative data, providing a reference for early intervention of the special population.

## Materials and methods

### Study population

This was a cross-sectional study using survey data from the National Survey of Children's Health (NSCH) 2016–2017 (14) and 2018–2019 (14). The NSCH aims to provide national and state-level data on the physical and emotional health of children in the United States. In this study, a total of 818 children who had been or were currently diagnosed with epilepsy were extracted from the NSCH 2016–2017 and 2018–2019. Children who were underweight and had missing study variables were excluded.

A total of 131,774 surveys were completed from 2016 to 2019 combined, 50,212 surveys were completed in 2016, 21,599 in 2017, 30,530 in 2018, and 29,433 in 2019. Survey data were weighted and adjusted to represent the national and state populations of non-institutionalized children aged 0–17 living in housing units in each state and nationally. The Overall Weighted Response Rate was 40.7% for 2016, 37.4% for 2017, 43.1% for 2018, and 42.4% for 2019 (15, 16).

Since we analyzed the de-identified public database released by the Data Resource Center for Child and Adolescent Health (DRC), our study was deemed exempt from the institutional review board at the researchers' institution. For more details on NSCH data availability and ethics, visit (www.childhealthdata.org).

### Sampling method

Since 2016, the NSCH has been administered online and by mail. Families with one or more children under 18 years old were randomly selected, and one child from each family was randomly selected as the respondent through the main topical questionnaire. The adult in the family who was familiar with children's physical condition and care was invited to complete a short screening questionnaire and a more detailed online or written 10–17 aged questionnaire (17).

### Diagnosis of epilepsy

The diagnosis of epilepsy was reported by respondents (primary caregiver) in the NSCH survey as “a doctor or other health care provider ever told you that this child has epilepsy or seizure disorder?” then “if yes, does this child currently have the condition?” then, “if yes, is it mild, moderate, or severe?” The severity of the seizures was reported by the primary caregiver.

### Outcome variable

The outcome variable was overweight and obesity in children 10–17 years. Parents or guardians were asked, “What is this child's current height?” And “How much does this child currently weigh?” Data on height and weight will not be published separately, but they were combined into body mass index (BMI), which was released. The weight status of children and youths was defined by the BMI percentile of age and gender. “Normal weight” status was determined by BMI between 5th and 84th percentile, “Overweight” status was at or greater than 85th to less than 95th percentile, and “Obesity” status was at or greater than 95th percentile (18).

### Social determinants

Social determinants included children's behavioral, and socioeconomic characteristics. According to the federal poverty level (FPL), the household poverty level was divided into four levels, below 100%, 100%–200%, 200%–400%, and above 400%. Parents' education was chosen by themselves from less than high school, high school or General Educational Development (GED), some college or technical school, college degree or higher.

Children's behavioral data included physical activity, afterschool activities, volunteering, screen time, and physical condition. (1) Physical activity was defined as “How many days did this child exercise, play a sport, or participate in physical activity for at least 60 min during the past week” (0 days, 1–3 days, 4–6 days, every day). (2) Afterschool activities were defined as “Did this child participant in a sports team or did he or she take sports lessons after school or on weekends?” (yes/no). (3) Volunteer was defined as “Any type of community service or volunteer work at school, church, or in the community” (yes/no). (4) Screen time: in the 2016–2017 questionnaire, there were two questions about screen time for children. “On an average weekday, about how much time does this child usually spend in front of a TV watching TV programs, videos, or playing video games?” And “spend with computers, cell phones, handheld video games, and other electronic devices, doing things other than schoolwork?” In the 2018–2019 questionnaire, NSCH merged the two problems and the question was “on most weekdays, about how much time did this child spend in front of a TV, computer, cellphone or other electronic device watching programs, playing games, accessing the internet or using social media? Do not include time spent doing schoolwork.” Response options are none, less than 1 h, 2–3 h, 4 or more hours (19). (5) Physical condition: In general, the parent would be asked, “how would you describe this child's health?” Parents choose from excellent, very good, good, fair, and poor and filled in the questionnaire as the basis for describing the physical conditions of the child.

### Data collection

Demographic data included sex (male/female), age [preadolescence (less than 13 years)/adolescence (13–17 years)], and race (Hispanic/non-Hispanic white/non-Hispanic black/non-Hispanic Others). Babies born before 37 weeks gestation were the major predictor of premature birth (20). So, the question “Was the child born more than 3 weeks before his or her due date?” (yes/no) was in the survey, which was used to assess premature birth. And, “how much did he or she weigh when born?” Answer in pounds and ounces or kilograms and grams to judge whether the birth weight was low. The NSCH dataset included parent reports of birth weight which was categorically recorded as not born with low weight (greater than 2,500 g), born with low weight (less than 2,500 g), and born with very low weight (less than 1,500 g) (Bureau).

Sleep duration would be examined “During the past week, how many hours of sleep did this child get on an average day?” (less than 6 h, 6 h, 7 h, 8 h, 9 h, 10 h, 11 or more hours). Children under 18 years of age should sleep at least 8 h per 24 h on a regular basis to promote optimal health according to the study, and younger children need more sleep (21).

Questions like “Lived with anyone who had a problem with alcohol or drugs (yes/no)” and “Does anyone living in your household use cigarettes, cigars, or pipe tobacco? if yes, does anyone smoke inside your home? (yes/no)” were questioned about the family environment.

### Statistical analysis

STATE and SAMPLE variables as strata, IDNUMR as the cluster, and NSCHWT as the weight were used in the analyses. The study population was divided into three groups (normal, overweight, and obesity) according to BMI and descriptive analysis was utilized. Counting data were described by cases and the weighted constituent ratio [*n* (weighted%)], and differences between groups were evaluated by the Chi-square test. Variables with significant differences between groups were included in the multivariate ordinal logistic regression model to explore the correlation between social determinants and overweight/obesity in children with epilepsy. Model 1 was the crude model, Model 2 adjusted for sex, age, and race, and Model 3 adjusted for sex, age, race, use of cigarets, cigars, or pipe tobacco inside, afterschool activity, and physical activity. Children with epilepsy were divided into three subgroups: those who were currently asymptomatic, mild, and moderate/severe symptoms to explore the correlation between socioeconomic factors and overweight/obesity. All the results presented are weighted. Statistical tests were conducted by bilateral tests, and *P *< 0.05 was statistically significant. Statistical analyses were performed by SAS v. 9.4 (SAS Institute, Cary, North Carolina) software. The proc power module in SAS v. 9.4 was used for power calculation.

## Results

### Description of the study population

**Figure 1:**
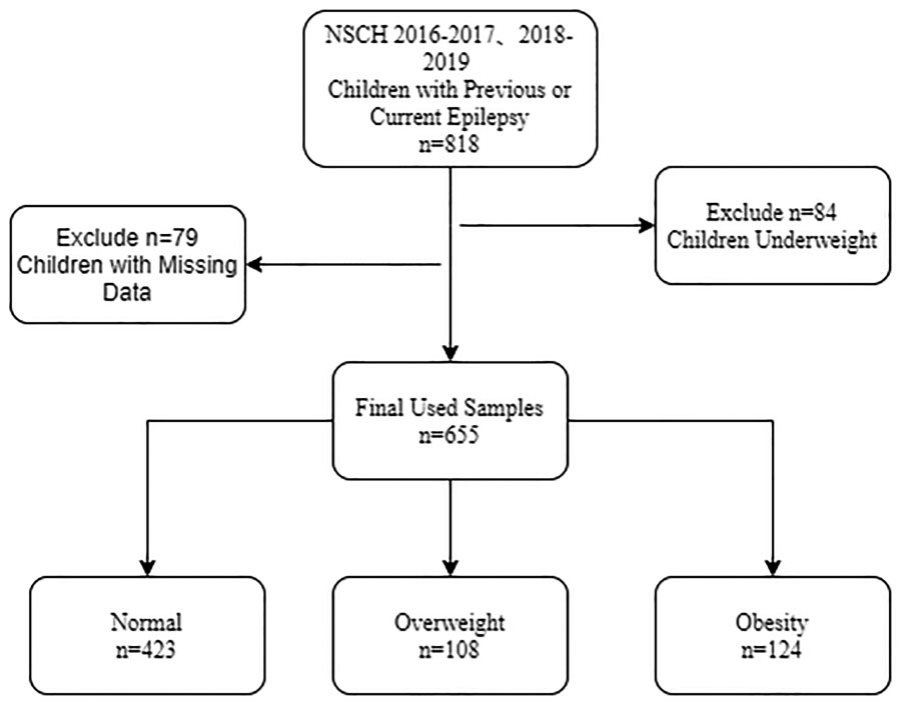
Study population.

There were 818 children with previous or current epilepsy in NSCH 2016–2017, 2018–2019, we excluded: (1) 84 underweight children (BMI < 5%); (2) 79 children with missing one or more data that we need for the following research. Finally, Data were analyzed on 655 children and youths with epilepsy aged 10–17, which was weighted to represent 247,823 children (Figure 1). We adopt the simple deletion method to deal with them due to these missing values in the data, then sensitivity analysis was conducted to compare the original data with the deleted data between groups. All the *P-*values were >0.05 in Supplementary Table S1, it showed no difference in observations that were excluded because of missing or invalid information.

Of the children included in the analysis, 423 were categorized into the normal weight group (BMI 5th–84th), 108 were categorized into the overweight group (BMI 85th–94th), and 124 were categorized into the obesity group (BMI ≥ 95th). There were 331 (52.64%) males and 324 (47.36%) females, 186 (34.15%) people under the age of 13, and 469 (65.85%) people between the ages of 13–17. Children and youths who do not currently have conditions [295 (42.84%)] accounted for the majority, followed by current conditions rated mild [191 (31.53%)] and current conditions rated moderate or severe [169 (25.63%)]. Characteristics of children with epilepsy in this study were shown in Table 1.

**Table 1:** Characteristics of children with epilepsy 10–17 years according to normal, overweight, and obesity status from the NSCH 2016–2017 and 2018–2019.

Characteristics	Total [*n* = 655 (weighted %)]	BMI group			*χ* ^2^	*P*
		Normal weight [*n* = 423 (weighted %)]	Overweight [*n* = 108, (weighted %)]	Obesity [*n* = 124 (weighted %)]		
Physical conditions, *n* (%)					16.693	0.012*
Excellent	186 (28.91)	142 (34.07)	29 (20.49)	15 (20.43)		
Very good	210 (33.53)	133 (35.58)	45 (41.85)	32 (24.37)		
Good	167 (24.12)	102 (22.70)	23 (27.78)	42 (25.79)		
Fair or poor	92 (13.43)	46 (7.64)	11 (9.88)	35 (29.41)		
Sex, *n* (%)					5.813	0.057
Male	331 (52.64)	218 (58.51)	53 (43.79)	60 (42.62)		
Female	324 (47.36)	205 (41.49)	55 (56.21)	64 (57.38)		
Age, *n* (%)					0.215	0.898
Preadolescence (<13)	186 (34.15)	119 (32.94)	34 (34.58)	33 (36.90)		
Adolescence (13–17)	469 (65.85)	304 (67.06)	74 (65.42)	91 (63.10)		
Race, *n* (%)					11.881	0.069
Hispanic	59 (16.77)	32 (13.31)	9 (10.39)	18 (28.43)		
Non-Hispanic White	483 (59.52)	319 (64.79)	83 (73.27)	81 (39.73)		
Non-Hispanic Black	46 (13.61)	27 (10.23)	6 (8.89)	13 (24.27)		
Non-Hispanic others	67 (10.10)	45 (11.67)	10 (7.45)	12 (7.57)		
Poverty level (FPL as reference), *n* (%)					24.960	<0.001*
Below 100%	54 (17.35)	29 (12.73)	8 (9.14)	17 (32.80)		
100%–200%	112 (21.56)	62 (17.54)	15 (20.64)	35 (31.88)		
200%–400%	187 (24.93)	114 (25.91)	34 (25.40)	39 (22.28)		
Above 400%	302 (36.16)	218 (43.82)	51 (44.82)	33 (13.05)		
Physical activity, *n* (%)					10.010	0.362
0 days	117 (19.18)	71 (15.86)	19 (21.82)	27 (26.02)		
1–3 days	277 (40.05)	166 (38.92)	52 (49.75)	59 (38.00)		
4–6 days	153 (26.01)	110 (29.72)	22 (16.25)	21 (21.77)		
Everyday	108 (14.76)	76 (15.51)	15 (12.17)	17 (14.21)		
Low birth weight, *n* (%)					4.128	0.393
Born with very low weight	24 (6.52)	16 (8.76)	4 (4.95)	4 (1.81)		
Born with low weight	65 (12.72)	35 (10.40)	14 (14.37)	16 (17.59)		
Not born with low weight	566 (80.76)	372 (80.84)	90 (80.68)	104 (80.60)		
Parents' education, *n* (%)					19.649	0.004*
Less than high school	47 (12.61)	20 (7.27)	8 (11.72)	19 (26.16)		
High school or GED	107 (18.41)	69 (16.95)	13 (10.82)	25 (25.77)		
College or technical school	194 (27.60)	120 (28.09)	32 (26.03)	42 (27.18)		
College degree or higher	307 (41.38)	214 (47.69)	55 (51.43)	38 (20.90)		
Premature birth, *n* (%)					0.142	0.932
Yes	110 (21.18)	67 (22.03)	20 (19.81)	23 (19.78)		
No	545 (78.82)	356 (77.97)	88 (80.19)	101 (80.22)		
Seizure severity, *n* (%)					10.637	0.033*
Do not currently have condition	295 (42.84)	200 (48.71)	50 (40.30)	45 (29.68)		
Current condition rated mild	191 (31.53)	131 (31.86)	27 (25.34)	33 (33.81)		
Current condition rated moderate or severe	169 (25.63)	92 (19.43)	31 (34.36)	46 (36.51)		
Afterschool activity, *n* (%)					3.006	0.224
Yes	463 (66.25)	308 (68.90)	80 (72.92)	75 (56.43)		
No	192 (33.75)	115 (31.10)	28 (27.08)	49 (43.57)		
Volunteer, *n* (%)					7.034	0.031*
Yes	305 (40.65)	201 (44.68)	55 (48.10)	49 (27.04)		
	350 (59.35)	222 (55.32)	53 (51.90)	75 (72.96)		
Use of cigarets, cigars, or pipe tobacco inside, *n* (%)					3.010	0.223
No	528 (79.38)	344 (79.50)	89 (86.92)	95 (75.34)		
Yes	127 (20.62)	79 (20.50)	19 (13.08)	29 (24.66)		
Screen time, *n* (%)					8.855	0.189
0–1 h/day	41 (6.71)	28 (8.08)	4 (2.89)	9 (5.22)		
2–3 h/day	216 (34.09)	145 (35.68)	36 (37.09)	35 (28.70)		
4 h/day or above	373 (55.52)	229 (51.38)	66 (58.83)	78 (64.01)		
Parent had a problem with alcohol or drugs, *n* (%)					2.387	0.305
Yes	106 (16.86)	74 (16.91)	11 (10.41)	21 (19.93)		
No	549 (83.14)	349 (83.09)	97 (89.59)	103 (80.07)		
Enough sleep, *n* (%)					3.335	0.190
Yes	461 (71.01)	303 (75.15)	79 (72.57)	79 (60.06)		
No	194 (28.99)	120 (24.85)	29 (27.43)	45 (39.94)		

FPL, federal poverty level; GED, General Educational Development.

* *P* < 0.05.

### Comparison of characteristics in different body mass index group

Results indicated statistically significant differences (*P *< 0.05) across several selected variables, physical conditions (*P *= 0.012), poverty status (*P *< 0.001), parents' education (*P *= 0.004), seizure severity (*P *= 0.033), and volunteer (*P *= 0.031) in Table 1. Furthermore, there was no difference in physical activity, birth data, parental alcohol or drug use, and other factors among normal, overweight, and obese children with epilepsy, *P *> 0.05.

### Association between socioeconomic status, daily habits, and obesity

**Figure 2:**
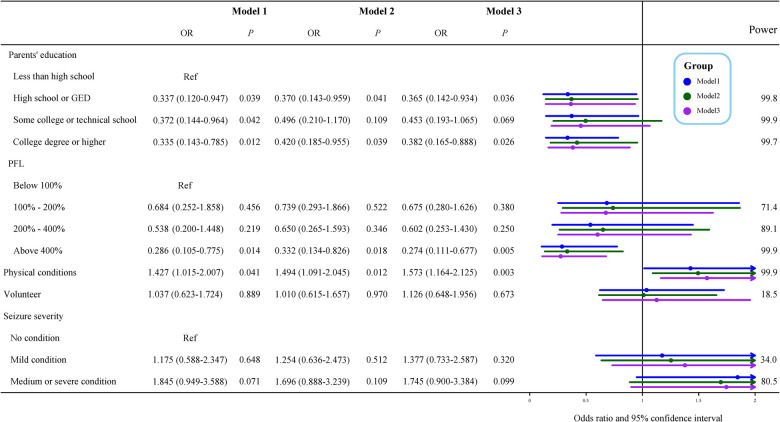
Multivariate ordinal logistic regression of characteristics for obesity. Model 1 was the crude model, Model 2 adjusted for sex, age, and race, and Model 3 adjusted for sex, age, race, use of cigarets, cigars, or pipe tobacco inside, afterschool activity, and physical activity. OR, odds ratio; Ref, reference; PFL, federal poverty level.

In Figure 2, parents' education was significantly correlated with obesity of the surveyed children in Model 3 (adjusted for sex, age, race, use of cigarets, cigars, or pipe tobacco inside, afterschool activity, and physical activity), odds ratio (OR) of high school or GED was 0.365 [95% confidence interval (CI), 0.142–0.934, *P *= 0.036], OR of college education or higher was 0.382 [(95% CI, 0.165–0.888), *P *= 0.026], less than high school as the reference. When the household poverty level was less than 100% FPL as the reference, greater than 400% FPL was negatively related to childhood obesity, the adjusted OR = 0.274 [(95% CI, 0.111–0.677), *P *= 0.005]. Children whose parents thought they were in poor or fair health were significantly correlated with obesity compared with those in good health, with the adjusted OR = 1.573 [(95% CI, 1.164–2.125), *P *= 0.003].

### Association between obesity and children with different severity of epilepsy

**Figure 3:**
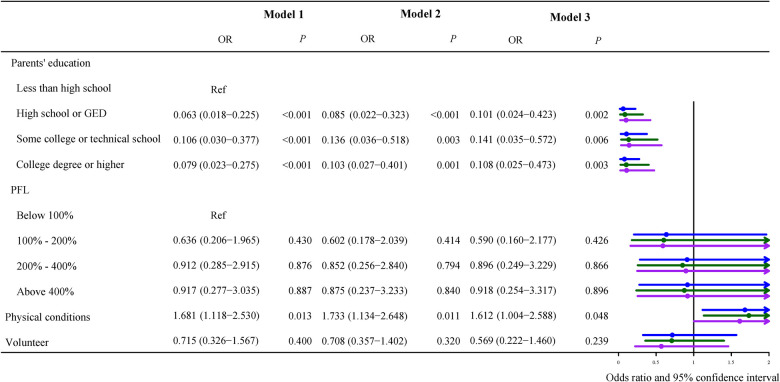
Odds ratios of currently asymptomatic children with epilepsy. Model 1 was the crude model, Model 2 adjusted for sex, age, and race, and Model 3 adjusted for sex, age, race, use of cigarets, cigars, or pipe tobacco inside, afterschool activity, and physical activity.

**Figure 4:**
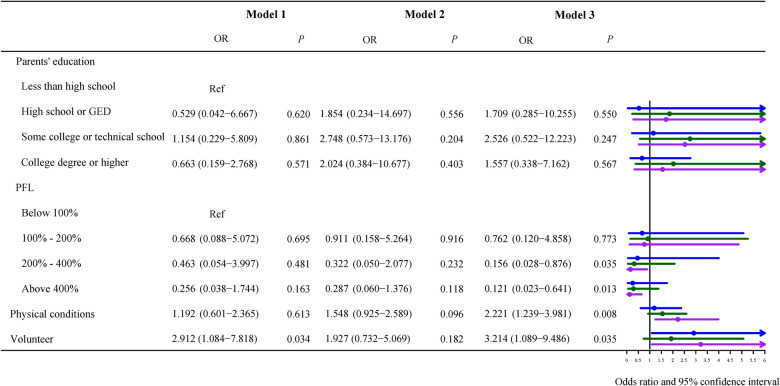
Odds ratios of children with mild symptoms of epilepsy. Model 1 was the crude model, Model 2 adjusted for sex, age, and race, and Model 3 adjusted for sex, age, race, use of cigarets, cigars, or pipe tobacco inside, afterschool activity, and physical activity.

**Figure 5:**
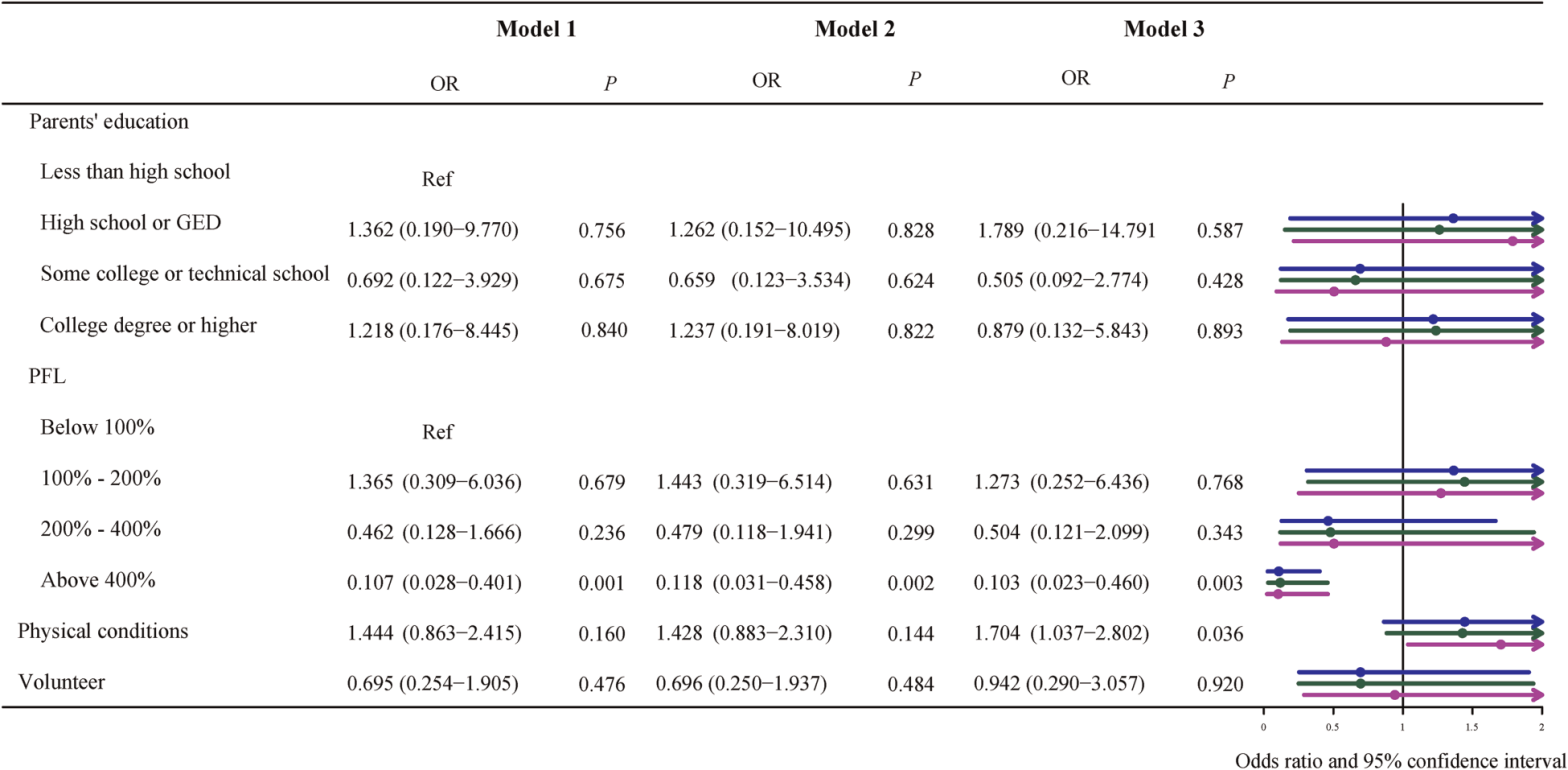
Odds ratios of children with moderate/severe symptoms of epilepsy. Model 1 was the crude model, Model 2 adjusted for sex, age, and race, and Model 3 adjusted for sex, age, race, use of cigarets, cigars, or pipe tobacco inside, afterschool activity, and physical activity.

Because our study population was children with epilepsy, we stratified them according to seizure severity, Figures 3–5 described results from the logistic regression for the three subgroups. Children with poor physical conditions reported by their parents were correlated with obesity in Model 3, OR of three subgroups (do not currently have the condition, current condition rated mild, current condition rated moderate or severe) were 1.612 [(95% CI, 1.004–2.588), *P *= 0.048], 2.221 [(95% CI, 1.239–3.981), *P *= 0.008], and 1.704 [(95% CI, 1.037–2.802), *P *= 0.036], respectively.

Among do not currently have condition children, their parents who graduated from high school or GED [OR = 0.101 (95% CI, 0.024–0.423), *P *= 0.002], some college or technical school [OR = 0.141 (95% CI, 0.035–0.572), *P *= 0.006], and college or higher [OR = 0.108 (95% CI, 0.025–0.473), *P *= 0.003] were all negatively correlated to childhood obesity with less than high school as the reference (Figure 3).

In the study population with current condition rated mild, poverty level less than 100% FPL as a reference, poverty level between 200% and 400% FPL [OR = 0.156 (95% CI, 0.028–0.876), *P *= 0.035] or above 400% FPL [OR = 0.121 (95% CI, 0.023–0.641), *P *= 0.013] were related to childhood obesity (Figure 4). Figure 4 demonstrated that not volunteering was also associated with obesity compared to children who volunteered [OR = 3.214, (95% CI, 1.089–9.486), *P *< 0.001].

For children with current conditions rated moderate or severe, poverty level less than 100% FPL was taken as the reference, as shown in Figure 5, there was a negative association between obesity and poverty level above 400% FPL, OR = 0.103 (95% CI, 0.023–0.460), *P *= 0.003.

## Discussion

Previous studies reported that epilepsy was associated with stigma (22). Young people with epilepsy are known to be at increased risk of impairment in their mental health, functioning, and social adjustment (23). And obesity is also a highly stigmatized condition in many societies, because of the belief of culpability (that patients are responsible for their weight) (24). In a school setting, weight-based bullying is one of the most common forms of peer harassment reported by students (25). Especially, epilepsy and obesity are also comorbid (5). Therefore, we took children and youths with epilepsy as the research participants to explore the correlation between social determinants and their overweight/obesity. The results showed that children who were in poor or fair physical conditions were significantly correlated with overweight/obesity compared with those who were in good physical conditions. Furthermore, we also found that parents' high education and high FPL were negatively correlated with overweight/obesity.

Studies have shown that parents with a college degree or higher (11) and high family income (26) may prevent children and youths from becoming overweight or obese. Our results were similar to those of studies not limited to children and youths with epilepsy, higher income was a lower predictor of overweight or obesity (27, 28). Indeed, we found that FPL was a related determinant of overweight or obesity, and a poverty level above 400% FPL was significantly corrected with overweight/obesity in children and youths with mild or moderate–severe epilepsy, as compared to a poverty level below 100% FPL. When parental graduation from high school or higher was related to overweight/obesity in previously diagnosed but now asymptomatic children and youths with epilepsy. Families with such socioeconomic determinants may provide children with the resources and opportunities to acquire and maintain healthier physical and mental lifestyles. Also, existing evidence suggested that socioeconomic factors may influence individuals' ability to make health decisions to varying degrees from microscopic factors such as epigenetic changes (29, 30). Epilepsy in children is common in low- and middle-income countries (30). These lines of evidence further supported the inverse associations of high parental education and high FPL with overweight/obesity that we found in children with epilepsy.

One of the reasons for the obesity epidemic is that people are increasingly using new media in their spare time instead of taking part in physical activity due to urbanization (31). Although studies have shown that children with epilepsy exercise less intensely than their non-epileptic siblings and are more likely to be overweight or obese (32). Our study showed that we found no clear link between obesity and exercise, other than volunteering. Children who were in poor or fair physical conditions were significantly correlated with overweight/obesity compared with those who were in good physical conditions. This suggested that young people with epilepsy were no longer subject to social external constraints as they used to be, conversely, children with epilepsy could be encouraged to engage in exercise as part of non-pharmacological therapy (32, 33).

To our knowledge, this was the first study to research multiple social determinants (household poverty level, parents' education, physical conditions, physical activity, etc.) simultaneously within a population sample of children with epilepsy. The results can provide the basis and direction for preventing obesity in children with epilepsy.

However, the study had some limitations. First, all data sources were from mail and online questionnaires, answered by parents of the children randomly selected, so there was a bias in the data. The main reason may be that the parents who filled in the questionnaire had a certain subjectivity in judging the real situation of their children. Parents' feedback on physical condition and seizure severity lacked clinical diagnostic evidence. Second, diet is often related to obesity, especially eating high-calorie junk food (34), and for children with anti-epileptic resistance, ketogenic diet therapy will also be given (35), but there is no information about children's personal eating habits in NSCH. Third, the study is lack information related to drugs or treatments for epilepsy of the question on receipt. Because the relationship between epilepsy and weight gain may result from the use of anti-epileptic drugs, such as valproate (VPA), gabapentin, carbamazepine (CBZ), vigabatrin, and pregabalin (PGB) (36). Despite these limitations, we provide a direction for the correlation between social determinants and overweight/obesity in children and youths with epilepsy, prospective studies with large samples are needed for further research.

## Conclusion

Parents with higher education and FPLs were negatively correlated to childhood obesity. Poor physical conditions were correlated with obesity in childhood epilepsy. It is worth noting that the findings of this study can be targeted at children with epilepsy in poor and low-income families to conduct health interventions on daily physical conditions. The long-term goal is to limit the incidence of obesity in children with epilepsy and reduce health expenditures.

## Data Availability

The original contributions presented in the study are included in the article/**Supplementary Material**, further inquiries can be directed to the corresponding author/s.
