# Supplementary material for: Exploring correlation between social determinants and overweight/obesity in children and youths with epilepsy
Source: Front Pediatr. 2022 Oct 21;10:897333. doi: 10.3389/fped.2022.897333 (PMC9634064; doi:10.3389/fped.2022.897333)
Supplement: Supplementary file 1 [file Table1.docx]

Supplement Table 1 Sensitivity analysis for excluded individuals

| Variables | Total (n=1389) | After (n=655) | Before (n=734) | χ2 | *P* |
| --- | --- | --- | --- | --- | --- |
| Physical conditions, n (%) |  |  |  | 1.196 | 0.755 |
| Excellent | 394 (28.79) | 186 (28.91) | 208 (28.68) |  |  |
| Very good | 446 (34.23) | 210 (33.53) | 236 (34.81) |  |  |
| Good | 352 (23.55) | 167 (24.12) | 185 (23.07) |  |  |
| Fair | 196 (13.43) | 92 (13.43) | 104 (13.43) |  |  |
| Poverty level (FPL as reference), n (%) |  |  |  | 5.203 | 0.160 |
| Below 100% | 117 (16.98) | 54 (17.35) | 63 (16.66) |  |  |
| 100% - 200% | 245 (22.10) | 112 (21.56) | 133 (22.57) |  |  |
| 200% - 400% | 389 (23.98) | 187 (24.93) | 202 (23.17) |  |  |
| Above 400% | 634 (36.93) | 302 (36.16) | 332 (37.59) |  |  |
| Physical activity, n (%) |  |  |  | 2.768 | 0.429 |
| 0 Days | 250 (18.85) | 117 (19.18) | 133 (18.56) |  |  |
| 1-3 Days | 588 (40.45) | 277 (40.05) | 311 (40.80) |  |  |
| 4-6 Days | 320 (26.40) | 153 (26.01) | 167 (26.74) |  |  |
| Everyday | 224 (14.30) | 108 (14.76) | 116 (13.90) |  |  |
| Low birth weight, n (%) |  |  |  | 1.180 | 0.555 |
| Born with very low weight | 51 (6.32) | 24 (6.52) | 27 (6.13) |  |  |
| Born with low weight | 135 (13.16) | 65 (12.72) | 70 (13.56) |  |  |
| Not born with low weight | 1178 (80.52) | 566 (80.76) | 612 (80.31) |  |  |
| Premature birth, n (%) |  |  |  | 2.472 | 0.116 |
| Yes | 229 (20.52) | 110 (21.18) | 119 (19.94) |  |  |
| No | 1,143 (79.48) | 545 (78.82) | 598 (80.06) |  |  |
| Seizure severity, n (%) |  |  |  | 1.170 | 0.558 |
| Do not currently have condition | 628 (43.05) | 295 (42.84) | 333 (43.23) |  |  |
| Current condition rated mild | 401 (31.89) | 191 (31.53) | 210 (32.20) |  |  |
| Current condition rated moderate/severe | 353 (25.06) | 169 (25.63) | 184 (24.57) |  |  |
| Afterschool activity, n (%) |  |  |  | 1.693 | 0.194 |
| Yes | 976 (67.02) | 463 (66.25) | 513 (67.69) |  |  |
| No | 406 (32.98) | 192 (33.75) | 214 (32.31) |  |  |
| Volunteer, n (%) |  |  |  | 0.137 | 0.712 |
| Yes | 641 (40.87) | 305 (40.65) | 336 (41.07) |  |  |
| No | 730 (59.13) | 350 (59.35) | 380 (58.93) |  |  |
| Use of cigarettes, cigars, or pipe tobacco inside, n (%) |  |  |  | 512 | 0.474 |
| No | 1,114 (78.80) | 528 (79.38) | 586 (78.30) |  |  |
| Yes | 268 (21.20) | 127 (20.62) | 141 (21.70) |  |  |
| Screen time, n (%) |  |  |  | 0.328 | 0.852 |
| 0-1hour/day | 138 (10.25) | 66 (10.39) | 72 (10.13) |  |  |
| 2-3hours/day | 459 (34.49) | 216 (34.09) | 243 (34.84) |  |  |
| 4hours/day or above | 786 (55.26) | 373 (55.52) | 413 (55.03) |  |  |
| Parent had a problem with alcohol or drugs, n (%) |  |  |  | 0.066 | 0.797 |
| Yes | 221 (17.05) | 106 (16.86) | 115 (17.22) |  |  |
| No | 1,154 (82.95) | 549 (83.14) | 605 (82.78) |  |  |
| Enough sleep, n (%) |  |  |  | 2.095 | 0.148 |
| Yes | 968 (69.84) | 461 (71.01) | 507 (68.83) |  |  |
| No | 415 (30.16) | 194 (28.99) | 221 (31.17) |  |  |
